# Left ventricular non-compaction cardiomyopathy with coronary artery anomaly complicated by ventricular tachycardia

**DOI:** 10.1186/s12872-017-0699-7

**Published:** 2017-10-16

**Authors:** Gustav Mattsson, Abdullah Baroudi, Hoshmand Tawfiq, Peter Magnusson

**Affiliations:** 1 Centre for Research and Development, Uppsala University/Region Gävleborg, SE-801 87 Gävle, Sweden; 2 Department of Medicine, Kiruna sjukhus, Region Norrbotten, SE-981 28 Kiruna, Sweden; 3 Cardiology Research Unit, Department of Medicine, Karolinska Institutet, SE-171 76 Stockholm, Sweden

**Keywords:** Cardiac imaging, Cardiac magnetic resonance, Cardiomyopathy, Coronary artery anomaly, Echocardiography, Heart failure, implantable cardioverter defibrillator, Non-compaction cardiomyopathy, Sudden cardiac death, Ventricular tachycardia

## Abstract

**Background:**

Non-compaction cardiomyopathy (NCCM) is characterized by prominent trabeculations, deep intertrabecular recesses, and a thick non-compacted endocardial myocardium. Prevalence in the general population remains unclear, but echocardiography series report 0.05%. During fetal development muscle fibers and trabeculae should compact into a solid myocardium and when this fails, NCCM occurs. The condition is genetic, even though acquired forms have been described. Worsening myocardial dysfunction may lead to heart failure and/or arrhythmias.

**Case presentation:**

A 52-year-old man presented with heart failure. The diagnosis of NCCM was confirmed after echocardiography and cardiac magnetic resonance tomography. Interestingly, the angiogram revealed a coronary anomaly, in which the circumflex artery rose aberrantly from the right coronary artery. Due to left ventricular ejection fraction being less than 35% despite optimal pharmacological therapy, an implantable cardioverter defibrillator (ICD) was implanted and four years later a ventricular tachycardia was terminated by antitachycardia pacing.

**Conclusion:**

We describe a case of NCCM with a concomitant coronary anomaly, in which systolic myocardial dysfunction developed. The ICD subsequently terminated a life-threatening ventricular arrhythmia, which supports risk stratification based on low ejection fraction and possibly coronary anomaly.

## Background

Non-compaction cardiomyopathy (NCCM) was first described in 1984 as a clinical entity and is a heterogeneous cardiomyopathy that can occur at any age [1, 2]. Congenital NCCM is caused by a defect in compaction of muscle fibers during fetal development, resulting in a spongiform myocardium. It appears that NCCM can also be acquired, for example due to athlete’s heart [3]. In a large hospital cohort the prevalence was 0.05%, and there seems to be an increased awareness among clinicians about NCCM [4]. Echocardiographic diagnosis is based on four criteria (Jenni criteria): absence of other cardiac abnormalities, end-systolic ratio between non-compacted endocardial myocardium and compacted epicardial myocardium of >2, localization of hyper-trabeculation to the apex/mid-inferior/mid-lateral areas, and color doppler showing blood flow from the ventricle into deep intertrabecular recesses without communication with coronary vessels [5]. Some patients with NCCM are asymptomatic, others develop heart failure with reduced systolic ejection fracture (EF). NCCM may increase the risk of ventricular tachycardia, and the risk of thromboembolism, likely due to reduced blood flow in the intertrabecular recesses [6]. NCCM exhibits familial clustering, with point prevalence of phenotype being 30% in first-degree relatives [7]. Genes encoding sarcomeric and cytoskeletal proteins have been implicated in NCCM, as well as genes previously linked to such disorders as hypertrophic cardiomyopathy, mitochondrial diseases, Barth syndrome, and myotonic dystrophy [8].

## Case presentation

**Fig. 1:**
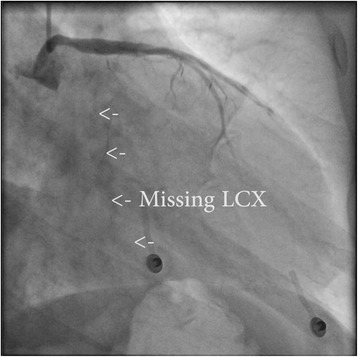
Coronary angiogram, left anterior oblique view with caudal angulation showing left coronary artery and left anterior descending artery, without any circumflex artery (missing LCX)

**Fig. 2:**
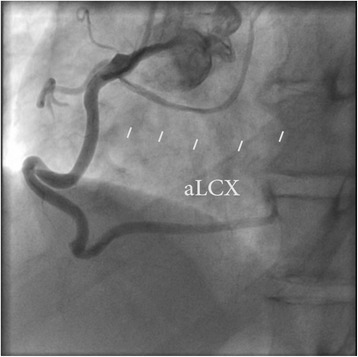
Coronary angiogram, right anterior oblique view with cranial angulation showing right coronary artery giving rise to a thin aberrant circumflex artery (aLCX)

**Fig. 3:**
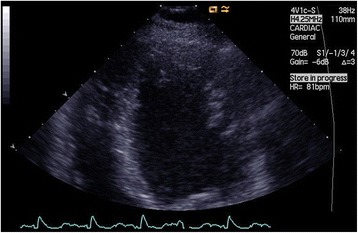
Transthoracic echocardiogram, end-diastole, apical four-chamber view showing prominent trabeculations in the left ventricular wall

**Fig. 4:**
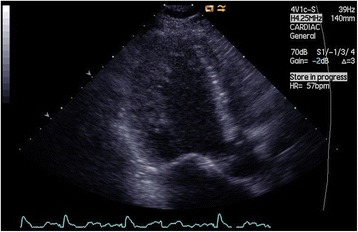
Transthoracic echocardiogram, systole, apical long-axis view showing a thick non-compacted myocardium in the left ventricular wall

**Fig. 5:**
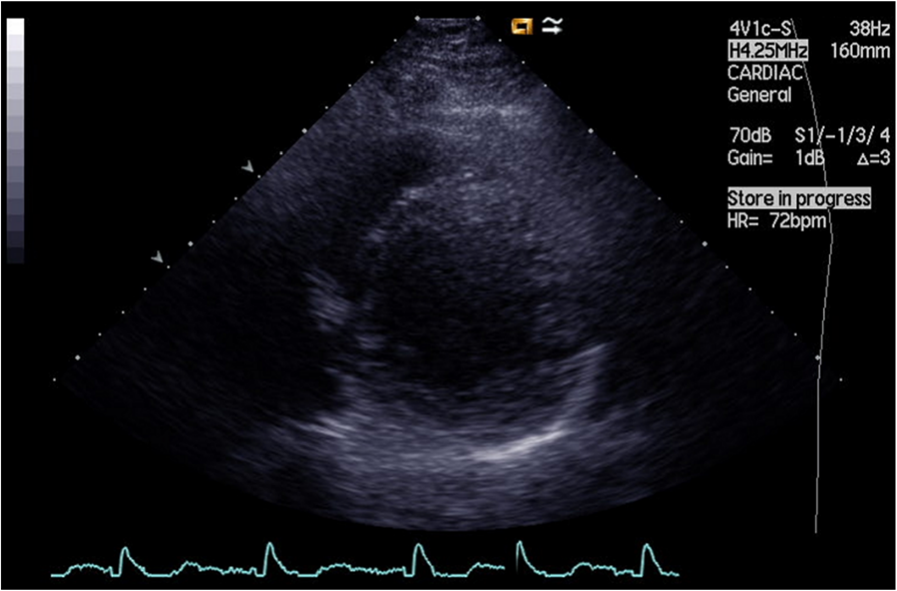
Transthoracic echocardiogram, end-diastole, parasternal short axis view showing hypertrabeculation of the inferolateral left ventricular wall

A 52-year-old man presented with dyspnea, chest discomfort, and palpitations upon exertion. His parents had confirmed ischemic heart disease. ECG at rest showed sinus rhythm, premature ventricular complexes and poor R-wave progression in precordial leads V_1_ to V_4_. The first echocardiography revealed general hypokinesia, predominantly in the anterior wall, thin walls without dilatation, EF around 35%, and high and pointy E-waves, indicative of a restrictive pattern. He reached 200 W on cycle ergometer exercise testing, but with frequent premature ventricular complexes. Angiography (Figs. 1 and 2.) showed no signs of coronary artery disease. Surprisingly, a coronary anomaly was revealed, in that the circumflex coronary artery (LCX) originated from the right coronary artery (RCA). At this point, cardiac magnetic resonance (CMR) imaging did not provide any further diagnostic clues. The situation was complicated by atrial fibrillation that was electrically converted but recurred at two-year follow-up. Another echocardiogram (Figs. 3, 4 and 5) was performed that for the first time raised suspicion of NCCM with EF around 30%. There were hypokinesia and deep intramyocardial recesses in the left ventricular wall. The ratio of non-compacted to compacted myocardium was 2.4 measured in the apical long-axis view, mid-inferior, at end-systole.

**Fig. 6:**
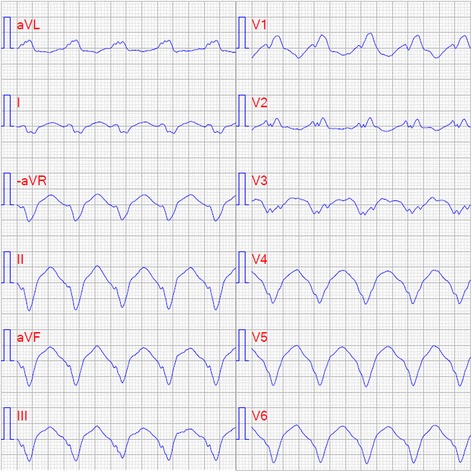
Electrocardiogram of monomorphic ventricular tachycardia, 200 beats per minute (paper speed 50 mm/s)

The patient was referred to a tertiary center for evaluation including right ventricular catheterization and endomyocardial biopsy that did not suggest any alternative diagnoses. Repeated echocardiograms confirmed the diagnosis of NCCM. An implantable cardioverter defibrillator (ICD) with a single lead (QRS < 120 ms) was offered to the patient for primary prevention of sudden cardiac death (SCD). Four years later, antitachycardia pacing terminated a life-threatening monomorphic ventricular tachycardia of 200 beats per minute (Fig. 6.). The patient has been followed for another three years and is on beta-blocker, angiotensin receptor blocker, and eplerenone therapy. In that time, he has had no further arrhythmias requiring ICD therapy and is New York Heart Association (NYHA) functional class II. The CARE guidelines were followed in the writing of this report.

## Discussion and conclusions

Clinical presentations of NCCM are heterogeneous and 35% have no hypertrophy or dilation of the ventricle. NCCM in the absence of arrhythmia seem to have similar survival rates as the general population [6]. Patients with NCCM with arrhythmia, are believed to have worse prognosis than patients with similar arrhythmias alone. Other forms of NCCM include dilated, hypertrophic, and restrictive pattern. NCCM typically affects the left ventricle, but biventricular and isolated right ventricular manifestations do occur [6]. There is no specific therapy for NCCM, but if heart failure is present this should be treated according to guidelines [9]. In our patient, metoprolol, enalapril, and eplerenone were titrated. The classic triad of NCCM consists of thromboembolism together with arrhythmia and heart failure. Anticoagulation is advocated if NCCM is associated with either atrial fibrillation, severe heart failure, confirmed thrombus, or previous thromboembolism [10]. In our patient, anticoagulation with warfarin was initiated due to atrial fibrillation and heart failure.

The Jenni echocardiography criteria are widely used but they can be difficult to validate and there is a lack of consensus about them [5]. CMR can provide additional value with borderline cases or in patients in whom differential diagnoses like hypertrophic cardiomyopathy are difficult to rule out. An end-diastolic ratio between non-compacted and compacted layers of >2.3 is considered diagnostic [11]. Late gadolinium enhancement in trabeculae is indicative of fibrosis and correlates with clinical severity [12].

This case report describes concomitant NCCM and aberrant LCX arising from the RCA, which occurs in 0.37% of angiograms [13]. Other coronary anomalies have previously been described together with NCCM, a single coronary artery of anomalous origin [14], as well as an anomaly including four arteries arising from the RCA with an LCX arising directly from the aorta [15]. How common coronary anomalies are in patients with NCCM is unknown, because the rarity of both conditions makes this difficult to study. Given the common pathogenesis of abnormalities in embryogenesis, an overrepresentation is plausible. The presence of coronary anomalies provides a challenge in risk stratification for SCD. The anomaly present in this case, aberrant LCX from the RCA, is generally not linked to an increased risk of SCD. In cases where the aberrant LCX travels between the aorta and the pulmonary artery, increased SCD has been reported, likely caused by compression or angulation of the artery [13].

The efficacy of ICD treatment in preventing death from arrhythmia is well established [16]. The European Society of Cardiology guidelines regarding SCD state that NCCM without further risk factors is not an indication for ICD. It is suggested to use the same criteria for risk stratification in NCCM as in non-ischemic, dilated cardiomyopathy [17]. ICDs should be offered to survivors of ventricular arrhythmias regardless of the underlying cardiac etiology. ICD should be offered as primary prophylaxis to NCCM patients with EF ≤35% and NYHA functional class II-III despite at least three months of optimal pharmacological therapy, and an otherwise reasonable life expectancy. Despite the limitations of EF estimation in individual patients, it still provides the best discriminator for risk stratification in most cardiomyopathy types [17, 18]. We believe that the current approach of risk stratification is advisable until more specific data of NCCM are available. The ICD in our patient terminated a life-threatening arrhythmia. This should encourage careful risk stratification in NCCM based on generalized knowledge from other cardiomyopathies.

## Abbreviations

CMR: Cardiac magnetic resonance
EF: Ejection fraction
ICD: Implantable cardioverter defibrillator
LCX: Circumflex coronary artery
NCCM: Non-compaction cardiomyopathy
NYHA: New York Heart Association
RCA: Right coronary artery
SCD: Sudden cardiac death
